# A case of erythrodermia from exacerbated psoriasis vulgaris due to treatment of acute hepatitis C

**DOI:** 10.1186/s12895-016-0042-5

**Published:** 2016-05-26

**Authors:** Eva Lemmenmeier, Barbara Gaus, Patrick Schmid, Matthias Hoffmann

**Affiliations:** Division of Infectious Diseases and Hospital Hygiene, Cantonal Hospital St.Gallen, Rorschacherstrasse 95, 9007 St.Gallen, Switzerland; Department of Dermatology, Cantonal Hospital St.Gallen, Rorschacherstrasse 95, 9007 St.Gallen, Switzerland

**Keywords:** Erythrodermia, Psoriasis, Acute hepatitis C, Pegylated-interferon-α

## Abstract

**Background:**

Skin side effects during interferon-alpha and ribavirin treatment are common, but autoimmune dermatosis triggered by interferon-alpha is rare. We describe a case of erythrodermia from exacerbated psoriasis during the treatment of acute hepatitis C with pegylated-interferon-alpha and ribavirin. The incidence of psoriasis in this circumstance is unknown and only 36 cases are described in the literature, of which only one describes an erythrodermic psoriasis flare.

**Case presentation:**

A 50-years old healthy white man presented with the complaints of headache, muscle pain, appetite loss, yellow skin complexion and fatigue. The laboratory results showed elevated liver enzymes above 50 times the upper limit of normal and a positive antibody test and RNA for hepatitis C. A screening test 6 months earlier was negative and therefore the diagnosis of an acute hepatitis C infection was most likely. In the absence of spontaneous clearance of the virus a therapy with pegylated- interferon-α and ribavirin was initiated. After 3 weeks the patient developed red scaly papular skin lesions that evolved despite treatment with prednisone to a severe erythrodermia. A skin biopsy showed typical signs for psoriasis vulgaris. Treatment with steroids was intensified and the hepatitis C therapy stopped. The patient achieved sustained virological response for hepatitis C, but psoriatic lesions were still present 6 months after treatment.

**Conclusion:**

Although autoimmune skin reactions under pegylated-interferon-α and ribavirin treatment are rare it is important to recognise them early to start an adequate treatment to guarantee hepatitis C treatment continuation.

## Introduction

Therapy of chronic hepatitis C (HCV) has changed from treatment with pegylated interferon-α (peg-INF-α) and ribavirin to combinations of direct acting agents (DAA’s) that enable interferon- or even ribavirin-free regimens. Interferon-free treatment regimens show cure rates >90 %, shorter treatment durations and less side effects. But access to DAA’s is limited to patients with chronic HCV infection and because of financial constraints even to patients with advanced liver damage (severe fibrosis or cirrhosis) in most countries [1, 2]. The effect of DAA therapies in acute HCV is not clear and data are lacking. Therefore treatment with peg-INF-α +/- ribavirin for 12–24 weeks is still the standard of care for acute HCV.

Treatment related skin reactions under interferon therapy are common (13 %), but usually they are mild and discontinuation of the drugs is not necessary [3, 4]. Injection site reactions are the most frequently encountered lesions. But also alopecia, eczematous drug reactions, fixed drug or lichenoid eruptions, pigmentation changes and sarcoidosis can be seen [3]. Seldom may an autoimmune skin disease such as psoriasis or lupus erythematodes be triggered by interferon treatment. We describe a case of erythrodermia from exacerbated psoriasis during the treatment of acute HCV with peg-INF-α and ribavirin.

## Case presentation

**Fig. 1:**
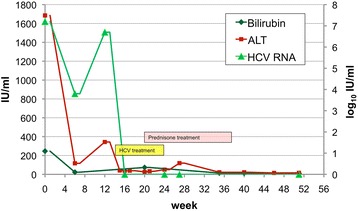
Liver enzymes, HCV-RNA over time

A 50 years old white man was sent to our outpatient clinic. He started to feel sick a month before with headache, muscle pain, appetite loss, yellow skin complexion and fatigue. His medical history was unremarkable except for two gout attacks years ago for which reason he was taking allopurinol for more than 5 years. He ceased taking it as soon as he felt sick. Alcohol consumption was three beers per day. Sexual history revealed intercourse with two women, homosexual contacts were negated. No intravenous or nasal drug use and no tattoos were reported. The clinical examination showed two red, erythrosquamous plaques of 10x10mm and 5x5mm in diameter in the décolleté and scleral icterus. Sonography of the liver revealed liver steatosis but no signs of fibrosis or cirrhosis. Liver enzymes were elevated above 50 times the upper limit of normal. HCV antibodies were positive. CMV, HIV and hepatitis A and B serology were negative, EBV serology consistent with past infection. The diagnosis of HCV genotype 1a was confirmed by HCV-RNA. Since a screening test for HCV was negative six months ago we considered this constellation despite a lacking transmission history as an acute HCV infection. For the following 12 weeks transaminases and HCV-RNA were monitored to follow a potential spontaneous HCV clearance (Fig. 1). Because the patient remained viraemic we initiated a treatment for acute HCV with 180 μg peg-INF-α weekly and 1000 mg ribavirin daily. Two weeks after treatment initiation HCV-RNA was undetectable (Fig. 1).

**Fig. 2:**
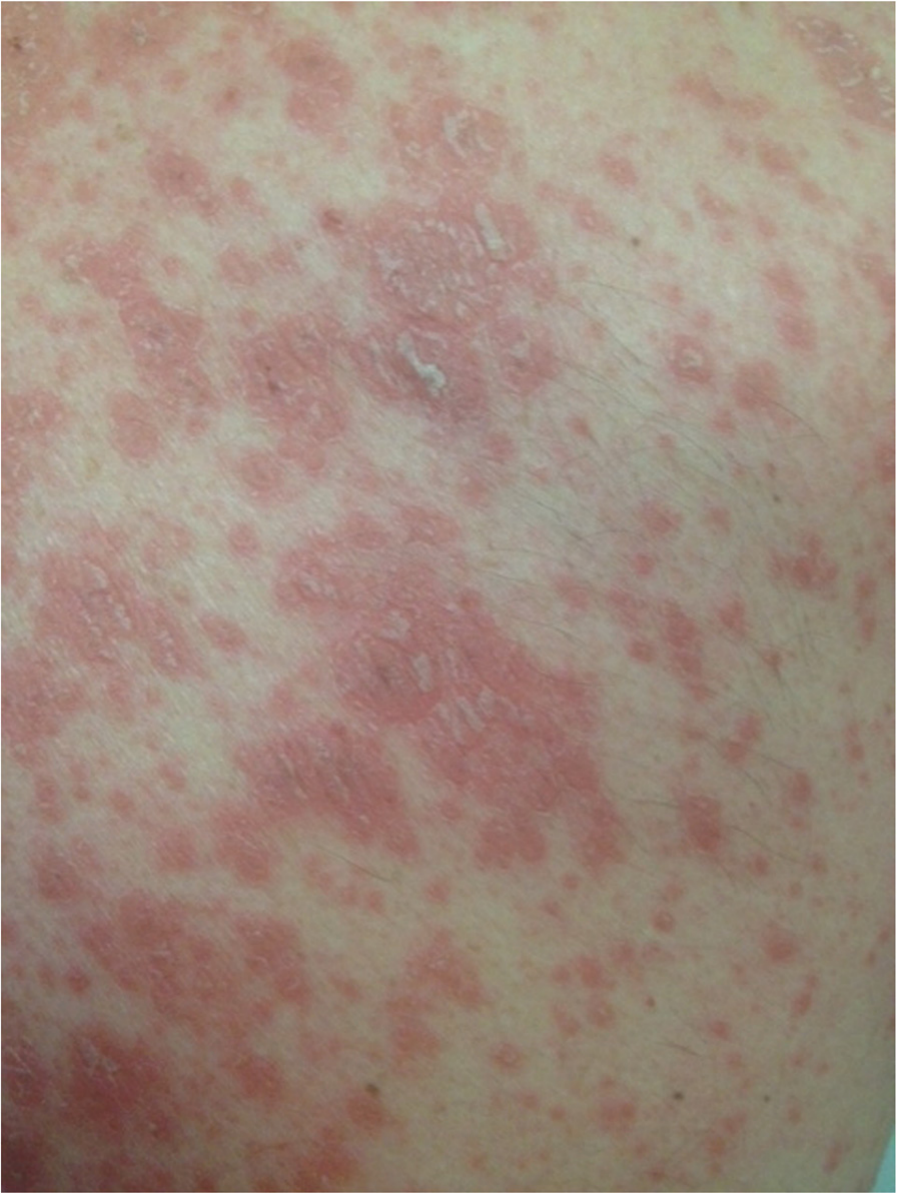
Psoriatic skin lesions at treatment week 6

**Fig. 3:**
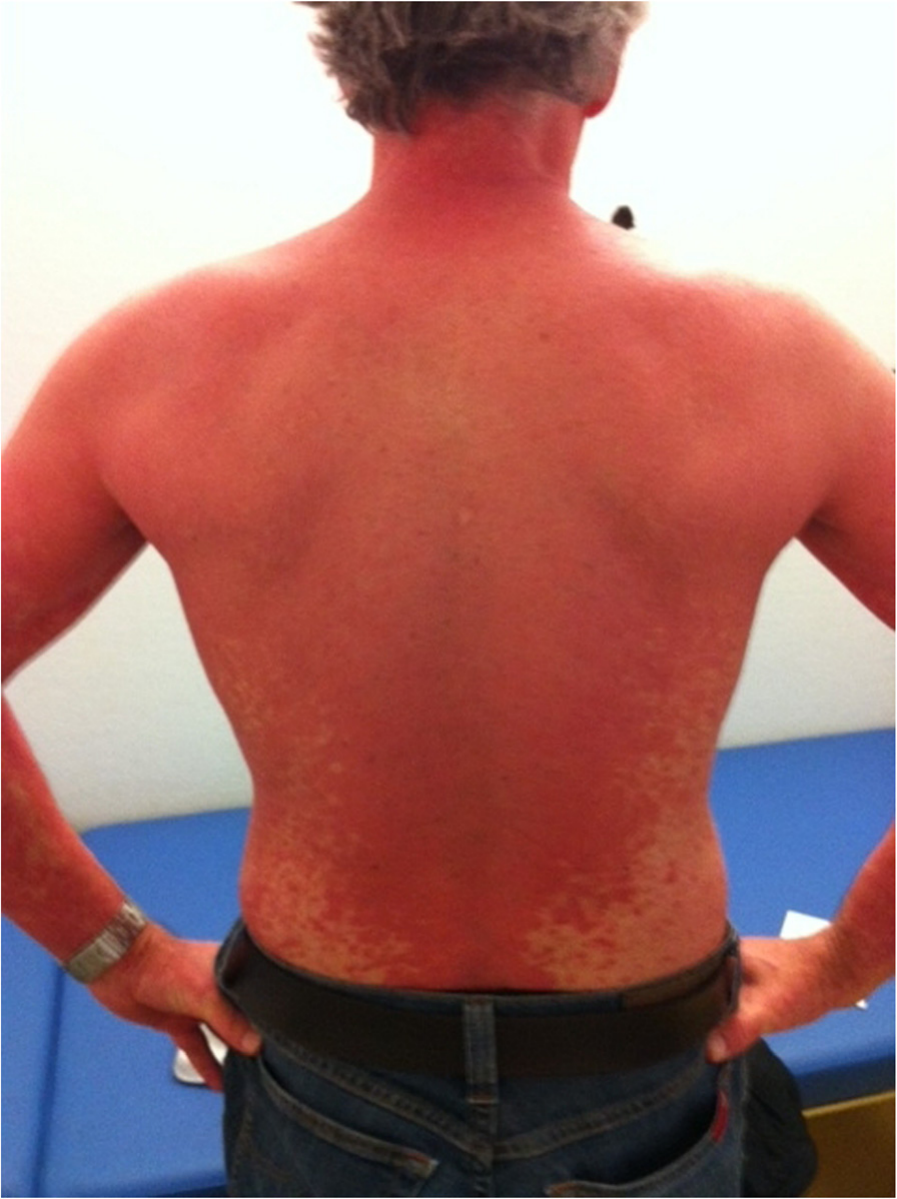
Erythrodermic psoriasis at treatment week 10

**Fig. 4:**
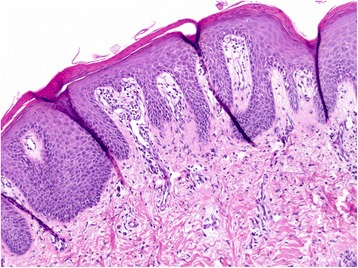
Histopathology of the skin biopsy

At treatment week 3 the patient developed itching red papules around 1 to 3 mm in diameter on the forearms and feet. Some of them were confluent to plaques and had a scaly aspect. Symptomatic treatment with cetirizine and hydrating lotion was started. Over the next weeks the exanthema was confluent and therapy with oral prednisone (0.7 mg/kg body weight) was added at treatment week 6 (Fig. 2). After an initial improvement the exanthema worsened again. Serological tests for HIV and syphilis were repeatedly negative and ribavirin was stopped at week 9. At week 10 the whole therapy was ceased because of increasing severe erythrodermia (Fig. 3). There were no oral lesions. One week after treatment cessation skin lesions aggravated further. A dermatologist was involved and a skin biopsy performed. The biopsy showed papillomatosis with vessel convolutes that reached the stratum corneum as well as neutrophiles in the dermis and parakeratosis consistent with psoriasis vulgaris (Fig. 4). Systemic prednisone doses were further increased to 1 mg/kg body weight (70 mg) and complemented with local whole body clobetasol propionate applications. HCV-RNA was not detectable at end of treatment and during follow up consistent with a sustained virological response (Fig. 1).

Over the next weeks after HCV treatment cessation prednisone could be tapered and replaced by 50 mg acitretin per day. 6 months after treatment termination the patient still had some psoriatic lesions and acitretin was maintained.

## Discussion

Even though new interferon-free DAA therapies are available for the treatment of chronic HCV, standard therapy for acute HCV remains peg-INF-α for 12–24 weeks (plus ribavirin under certain conditions e.g. HIV/HCV-co-infection) [5]. Data of treatment for acute HCV with DAA’s is lacking. Only one study in HIV/HCV co-infected men who have sex with men showed an increased sustained virological response at week 12 in patients treated with combination therapy of telaprevir/peg-INF-α/ribavirin compared to peg-INF-α/ribavirin alone [6]. Additionally since access to interferon-free treatment is limited to patients with advanced liver damage because of high costs even treatment of chronic HCV with peg-INF-α has still to be considered in rare cases.

Erythrodermic psoriasis is a rare but a severe and disabling variant of psoriasis vulgaris [7]. The incidence of psoriasis exacerbation by peg-INF-α therapy is unknown, but several case reports were published [8–11]. One small uncontrolled explorative study with 28 patients reported an occurrence of psoriasis in 2 % (3/28) [12]. A case compilation by Afshar et al. found 36 cases of INF-α associated psoriasis flares [8]; most cases were classic plaque-type psoriasis vulgaris and only one patient showed erythrodermic psoriasis. Most psoriasis flares happened with an average delay of 1.6 months after interferon treatment initiation but with a high inter-individual variation (1 week -7 months) [8]. Our patient’s psoriatic flare was early in the treatment course starting at week three and showing the full picture of erythrodermic psoriasis at week 10. In our case treatment had to be stopped. This is usually not necessary since mostly psoriatic skin side effects are not severe and diminish after stopping therapy [8]. Retrospectively our patient had mild psoriatic lesions already at the initial diagnosis of HCV infection. Since they seemed well controlled without specific treatment at the time HCV treatment with peg-INF-α was not contraindicated [13].

The report highlights the importance of a throughout patient history and clinical examination at hepatitis C treatment initiation and – most importantly – in regular intervals thereafter. It is of utmost importance to recognise autoimmune processes like psoriasis early to be able to initiate an adequate therapy early and avoid hepatitis C treatment interruptions. It should be noted that a well-controlled psoriasis is not a contraindication to a treatment with peg-INF-α and ribavirin since combination therapies with immunosuppressive treatment seem safe [14].

## Abbreviations

CMV, Cytomegalovirus; DAA, direct acting agent; EBV, Ebstein-Barr virus; HCV, hepatitis C; HIV, Human immunodeficiency virus; Peg-INF-α, pegylated interferon-α; RNA, Ribonucleic acid.

## References

[1] Kohli A, Shaffer A, Sherman A, Kottilil S (2014). Treatment of hepatitis C: a systematic review. JAMA.

[2] Cortez KJ, Kottilil S (2015). Beyond interferon: rationale and prospects for newer treatment paradigms for chronic hepatitis C. Ther Adv Chronic Dis.

[3] Mistry N, Shapero J, Crawford RI (2009). A review of adverse cutaneous drug reactions resulting from the use of interferon and ribavirin. Can J Gastroenterol.

[4] Patrk I, Morović M, Markulin A, Patrk J (2014). Cutaneous reactions in patients with chronic hepatitis C treated with peginterferon and ribavirin. Dermatol Basel Switz.

[5] European Association for the Study of the Liver (2015). EASL Recommendations on Treatment of Hepatitis C 2015. J Hepatol.

[6] Fierer DS, Dieterich DT, Mullen MP, Branch AD, Uriel AJ, Carriero DC (2014). Telaprevir in the treatment of acute hepatitis C virus infection in HIV-infected men. Clin Infect Dis.

[7] Hawilo A, Zaraa I, Benmously R, Mebazaa A, El Euch D, Mokni M (2011). Erythrodermic psoriasis: epidemiological clinical and therapeutic features about 60 cases. Tunis Médicale.

[8] Sharifi AH, Fakharzadeh E, Zamini H, Haj-Sheykholeslami A, Jabbari H (2012). Exacerbation of Skin Lesions in a 50 year old Man with Psoriasis during Treatment by Pegylated Interferon. Middle East J Dig Dis.

[9] Horev A, Halevy S (2009). New-onset psoriasis following treatment with pegylated interferon-alpha 2b and ribavirin for chronic hepatitis C. Isr Med Assoc J IMAJ.

[10] Dag MS, Oztürk ZA, Yılmaz N, Cam H, Kadayıfçı A (2013). Peginterferon alfa related psoriasis in a patient with acute hepatitis C and review of the literature. Wien Klin Wochenschr.

[11] Afshar M, Martinez AD, Gallo RL, Hata TR (2013). Induction and exacerbation of psoriasis with Interferon-alpha therapy for hepatitis C: a review and analysis of 36 cases. J Eur Acad Dermatol Venereol JEADV.

[12] Li Z, Zhang Y, An J, Feng Y, Deng H, Xiao S (2014). Predictive factors for adverse dermatological events during pegylated/interferon alpha and ribavirin treatment for hepatitis C. J Clin Virol.

[13] Bartalesi F, Salomoni E, Cavallo A, Corti G, Pimpinelli N, Bartoloni A (2013). Chronic hepatitis C virus hepatitis and psoriasis: no longer a contraindication to interferon use in the era of biological agents?. Scand J Infect Dis.

[14] Behnam SE, Hindiyeh R, Fife DJ, Jeffes EW, Wu JJ (2010). Etarnercept as prophylactic psoriatic therapy before interferon-alpha and ribavirin treatment for active hepatitis C infection. Clin Exp Dermatol.

